# Is Still's Disease an Autoinflammatory Syndrome?

**DOI:** 10.1155/2012/480373

**Published:** 2012-04-26

**Authors:** Linda Rossi-Semerano, Isabelle Koné-Paut

**Affiliations:** Department of Paediatrics and Paediatric Rheumatology, Bicêtre Hospital, National Reference Centre for Autoinflammatory Diseases, 78 rue du Général Leclerc, Le Kremlin Bicêtre, 94270 Paris, France

## Abstract

Systemic juvenile idiopathic arthritis (sJIA), formerly called Still's disease, is officially classified as a subset of juvenile idiopathic arthritis (JIA). Beside arthritis, it is characterized by prominent systemic features and a marked inflammatory response. Even if it is still included in the group of juvenile arthritides, sJIA is set apart from all the other forms of JIA. This disorder has markedly distinct clinical and laboratory features suggesting a different pathogenesis. sJIA does not show any association with HLA genes or with autoantibodies and is characterised by an uncontrolled activation of phagocytes with hypersecretion of IL-1 and IL-6. Based on clinical and laboratory features, as well as on new acquisitions on the pathogenesis, it seems evident that sJIA is an autoinflammatory disease related to abnormality in innate immune system. The new insights on the pathogenesis of sJIA have therefore dramatically changed the approach to treatment, with the development of targeted treatments (anti-IL-1 and anti-IL-6 agents) more effective and safer than earlier medications.

## 1. Introduction

Juvenile idiopathic arthritis (JIA) is the most common chronic rheumatic disease of childhood. It is a heterogeneous disease of unknown aetiology encompassing different forms of arthritis, which begins before the age of 16 years and persists for more than 6 weeks. JIA classification [1] is based on the number of joints involved during the first 6 months of disease and on the extra-articular involvement.

Most JIA subsets are characterized by female predominance, prominent arthritis, various degrees of biological inflammation, a strong susceptibility associated with some HLA class II antigens, and an overt or suspected autoimmunity, for example, antinuclear antibodies (ANA) rheumatoid factor (RF) and anticyclic-citrullinated peptide (anti-CCP) antibodies. Dramatic response to anti-TNF*α* treatments [2] is an important feature, which supports the role of the adaptative immunity in generating chronic inflammation.

## 2. Still's Disease as a Subset of Juvenile Idiopathic Arthritis

sJIA was officially classified as a subset of JIA, and the presence of at least one active synovitis was mandatory to support the diagnosis, even if some patients do not present arthritis at disease onset [3]. Moreover, sJIA can have a highly variable outcome, and a monocyclic course with minimal or absent articular complications was reported in about 50% of 56 cases [4]. Other differences with the other subtypes of JIA include an equal sex ratio, marked systemic features with spiking fever, a salmon-colored evanescent rash that comes and goes with fever, serositis, and the absence of autoantibodies.

The recognition of a group of rare diseases, the autoinflammatory diseases (AIDs) appearing to be primarily inflammatory in nature because of their periodicity, strong associations with exogenous triggering events, and lack of associations with class II MHC haplotypes, brought some evidence to look at sJIA as a distinct entity from other subtypes of JIA. Recent advances in understanding the role of IL-1 in the pathogenesis of sJIA brought strong arguments to consider the disease as autoinflammatory rather than autoimmune.

## 3. sJIA as Autoinflammatory Disease (AID)

**Figure 1:**
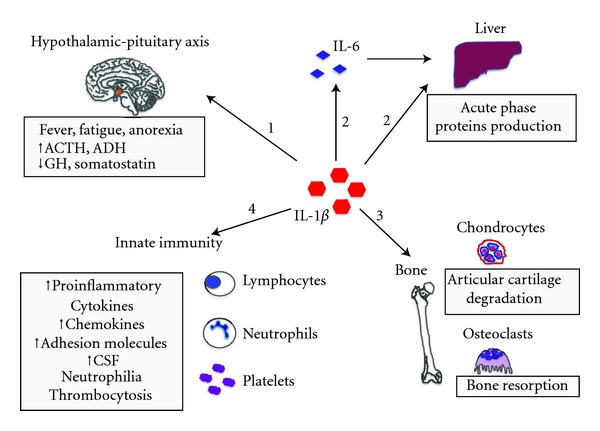
Main effects of IL-1. (1) The action on the hypothalamic-pituitary axis influences the production of the following hormones: adrenocorticotropic hormone (ACTH), growth hormone (GH), vasopressin or antidiuretic hormone (ADH), and somatostatin. IL-1 is responsible for the constitutional symptoms in IL-1-dependent diseases (fever, fatigue, anorexia, and growth delay). (2) Liver synthesis and secretion of acute phase proteins (both by direct IL-1 action and via IL-6 induction). (3) Osteoclasts activation and matrix metalloproteinases (MMPs) synthesis by chondrocytes, resulting in bone resorption and cartilage degradation, respectively. (4) Innate immune system cells activation and proliferation, enhanced gene transcription of proinflammatory molecules (inducible nitric oxide synthase (iNOS), cyclo-oxygenase 2 (COX2), and phospholipase A2), proinflammatory cytokines, adhesion molecules, and colony-stimulating factors (CSF).

AIDs are a large group of diseases affecting primarily the innate immune system. Despite their different molecular mechanisms, they are all characterized by an inappropriate activation of the phagocytes, the key cells of innate immune system. They have in common an overproduction of IL-1*β*, a prototype of proinflammatory cytokine having pleiotropic properties (Figure 1). Typical clinical manifestations of AIDs are recurrent, seemingly unprovoked, inflammatory attacks of fever with skin involvement, serositis, and arthritis. Laboratory examinations during fever attacks show a prominent acute inflammatory response characterised by a marked leukocytosis (neutrophilia), increased CRP, and serum amyloid protein (SAA). Unlike patients with autoimmune diseases, patients with AID lack high-titers autoantibodies and antigen-specific T cells.

Historically, AIDs comprised rare disorders of Mendelian inheritance like cryopyrin associated periodic syndrome (CAPS) (autosomal dominant) and familial Mediterranean fever (autosomal recessive). With time, other diseases, less rare and of multifactorial inheritance, like pFAPA syndrome (periodic fever, aphtous, pharyngitis, and adenitis), have joined the group. AIDs uniquely respond to specific IL-1*β* blockade unlike autoimmune diseases that respond dramatically to anti-TNF*α* treatments. Over the past decade, a growing number of systemic inflammatory disorders have been placed into the group of AIDs given their response to anti-IL-1 drugs, including sJIA and adult Still's disease (AoSD) [5].

## 4. Clinical Characteristics of sJIA

sJIA represents 10–15% of all JIA, with a broad peak of onset between 0 and 5 years of age, with 2 years being the most common [3], and an equal sex ratio. It is called Still's disease (AoSD) when it occurs in patients over the age of 16. AoSD is less common than sJIA but the disease features are the same, even severe arthritis occurs exceptionally. Therefore, sJIA and AoSD likely represent a continuum of the same disease entity [6].

SJIA is defined by [1] the presence of arthritis in one or more joints associated with spiking fever (a typically daily high fever with spike in the evening) persisting for a minimum of 15 days, with at least one of the following manifestations: skin rash (evanescent, nonfixed erythematous rash that accompanies fever spikes), generalized lymphadenopathy, hepatomegaly and/or splenomegaly, or serositis (pleuritis or pericarditis).

None of the clinical signs is specific to sJIA, especially at presentation, and differential diagnosis can be difficult (bacterial and viral infections, malignancy, and other rheumatic diseases). Moreover, arthritis may be absent at onset and can develop during disease course, usually progressing to a polyarticular and symmetrical involvement.

The disease course can be highly variable. It can be monocyclic, polycyclic with relapses followed by intervals of remission, or unremitting, leading about half of the patients to a chronic destructive arthritis representing the major long-term problem.

SJIA shows a strong association with macrophage activation syndrome (MAS), a form of reactive hemophagocytic lymphohistiocytosis (HLH), characterised by an uncontrolled activation of well-differentiated macrophages releasing a high amount of proinflammatory cytokines, particularly IL-18, which belongs to the IL-1 family. MAS is a severe, potentially life-threatening disorder, and clinically characterized by fever, hepatosplenomegaly, lymphadenopathy, neurologic dysfunction, and coagulopathy. Some studies suggest that up to 50% of sJIA patients might have occult MAS [7, 8]. Heterozygous mutations in genes involved in HLH have been described in some subsets of SoJIA patients and might play a role in the development of MAS [9]. Specific criteria for sJIA-associated MAS have been recently proposed [10]. Interestingly, MAS has been recently included as an individual group of autoinflammatory diseases in an updated classification proposed by Masters et al. [5].

## 5. Laboratory

Laboratory tests show a marked inflammatory response with leukocytosis (neutrophilia), thrombocytosis, high C-reactive protein (CRP) and erythrocyte sedimentation rate (ESR). In most cases a microcytic anaemia related to prominent inflammation is detected.

Markedly distinct clinical and laboratory features of sJIA suggest a different pathogenesis from the other forms of JIA. Oligopolyarticular form of JIA (the most frequent form) is an antigen-driven lymphocyte-mediated autoimmune disease with abnormality in the adaptive immune system [11]. On the other side, sJIA does not show any association with HLA genes nor with autoantibodies and is characterised by an uncontrolled activation of phagocytes. These features are all consistent with what is observed in autoinflammatory diseases [12–14].

## 6. Pathogenesis of sJIA

Phagocytes including monocytes, macrophages, and neutrophils are the principal activated cells during the early course of sJIA. It has been shown that these cells secrete very high levels of pro-inflammatory cytokines (IL-1, IL-6, IL-18) as well as proinflammatory proteins (S100A8, S100A9, and S100A12) [15–17].

## 7. The Role of IL-1 in sJIA

The discovery of an important role of IL-1 in the etiopathogenesis of sJIA came from studies that analyzed gene transcription patterns in peripheral blood mononuclear cells (PBMCs) from healthy individuals, incubated with serum from patients with active disease. Serum from sJIA patients can induce the transcription of IL-1*β* and various IL-1-related genes in healthy PBMCs [12]. Activated monocytes from sJIA patients secreted higher amounts of IL-1 (16-fold greater) compared to monocytes from healthy controls. The role of IL-1 was confirmed by studies showing the efficacy of anakinra, a recombinant anti-IL-1 receptor antagonist [12, 18]. Similar results were also reported for the related disorder AoSD [19].

Several open-label studies reported the clinical efficacy of anakinra in sJIA patients, with response rates around 50% [12, 19, 20]. More recently, a randomised double-blind placebo-controlled study in sJIA reported similar clinical response rates and normalization of the expression of genes involved in IL-1*β* regulation [18]. Despite a good short-term clinical control, most patients experienced loss of efficacy with ongoing anakinra treatment. The latter might be due to patients selection, being more likely observed in patients with long-standing refractory disease or in those with polyarticular involvement. Accordingly, in a case series of 22 pediatric sJIA patients, a low joint count and high blood PMN were positive predictors of clinical response to anakinra [20]. 

Nigrovic et al. recently reported a retrospective study with anakinra as first-line treatment in sJIA, with 59% of patients undergoing remission [21]. Early introduction of anakinra hindered arthritis relapse in 90% of patients. Further studies on the efficacy and safety of anakinra as first-line treatment are needed.

The response to anakinra can therefore identify two subsets of sJIA patients, one with dramatic response similar to that observed in (CAPS) [20, 22] and the other resistant or with an intermediate response [18, 21].

Preliminary results on canakinumab and rilonacept treatment showed a high efficacy in sJIA patients [23, 24].

## 8. The Role of IL-6

The levels of IL-6 are markedly elevated in the serum and synovial fluid of sJIA compared to other subtypes of JIA. Circulating levels are increased during the peak of fever and correlate with clinical activity, systemic features as thrombocytosis and microcytic anemia, growth retardation, osteopenia, and the extent and severity of joint involvement [16, 25, 26]. It has been suggested that polymorphisms involving the promoter elements and genes encoding IL-6 may contribute to the overproduction of IL-6 in sJIA [27, 28].

The major pathogenic role of IL-6 has been confirmed by the marked efficacy of tocilizumab, a monoclonal antibody targeting the IL-6 receptor, in reducing systemic features like fever and rash and improving inflammatory arthritis [29, 30].

## 9. The Role of IL-18

IL-18 is a member of the IL-1 cytokine superfamily, produced mainly by monocytes macrophages in response to viral or bacterial stimuli, which may contribute to the inflammatory process. sJIA, AoSD, and MAS are all characterised by extremely high IL-18 serum levels [31, 32]. Some reports have recently shown its elevation during sJIA flares and during the active phase of MAS [31]. A defective phosphorylation of IL-18 receptor has been reported in sJIA patients [33].

Based on clinical and laboratory features as well as on the new acquisitions on the pathogenesis, it seems evident that sJIA is an autoinflammatory disease related to abnormality in innate immune system. The marked activation of innate immune system responsible for the multisystem inflammation and the lack of any consistent association with HLA antigens or autoantibodies allow to consider sJIA as an autoinflammatory disease. This hypothesis is further confirmed by the response to anti-IL-1 and IL-6 agents.

Nevertheless, even if there is evidence for IL-1*β* deregulation on sJIA, further fundamental experiments are needed to explain whether this is due to intrinsic abnormalities in caspase-1 activation or it is rather linked to extrinsic mechanisms involving, for example, the TLRs and NF-KB activation pathway. From a clinical point of view, not every patient may respond completely to IL-1 inhibition, and the presence of polyarthritis is associated to worse results. The development of arthritis could be associated to a cytokine shift towards IL-6 and TNF*α*. The response of sJIA to tocilizumab (anti-IL-6 agent) is not contradictory because the IL-1*β* induces downstream secretion of IL-6, which shares many biological properties with IL-1.

The new acquisitions on the pathogenesis of sJIA have therefore dramatically changed its management, with the development of targeted therapy more effective and safer than earlier medications.

## References

[1] Petty RE, Southwood TR, Manners P (2004). International league of associations for rheumatology classification of juvenile idiopathic arthritis: second revision, edmonton, 2001. *Journal of Rheumatology*.

[2] Quartier P, Taupin P, Bourdeaut F (2003). Efficacy of etanercept for the treatment of juvenile idiopathic arthritis according to the onset type. *Arthritis & Rheumatism*.

[3] Behrens EM, Beukelman T, Gallo L (2008). Evaluation of the presentation of systemic onset juvenile rheumatoid arthritis: data from the Pennsylvania Systemic Onset Juvenile Arthritis Registry (PASOJAR). *Journal of Rheumatology*.

[4] Gurion R, Lehman TJA, Moorthy LN (2012). Systemic arthritis in children: a review of clinical presentation and treatment. *International Journal of Inflammation*.

[5] Masters SL, Simon A, Aksentijevich I, Kastner DL (2009). Horror autoinflammaticus: the molecular pathophysiology of autoinflammatory disease. *Annual Review of Immunology*.

[6] Luthi F, Zufferey P, Hofer MF, So AK (2002). “Adolescent-onset Still's disease”: characteristics and outcome in comparison with adult-onset Still's disease. *Clinical and Experimental Rheumatology*.

[7] Behrens EM, Beukelman T, Paessler M, Cron RQ (2007). Occult macrophage activation syndrome in patients with systemic juvenile idiopathic arthritis. *Journal of Rheumatology*.

[8] Bleesing J, Prada A, Siegel DM (2007). The diagnostic significance of soluble CD163 and soluble interleukin-2 receptor *α*-chain in macrophage activation syndrome and untreated new-onset systemic juvenile idiopathic arthritis. *Arthritis & Rheumatism*.

[9] Vastert SJ, van Wijk R, D’Urbano LE (2009). Mutations in the perforin gene can be linked to macrophage activation syndrome in patients with systemic onset juvenile idiopathic arthritis. *Rheumatology*.

[10] Davì S, Consolaro A, Guseinova D (2011). An international consensus survey of diagnostic criteria for macrophage activation syndrome in systemic juvenile idiopathic arthritis. *Journal of Rheumatology*.

[11] Lin YT, Wang CT, Gershwin ME, Chiang BL (2011). The pathogenesis of oligoarticular/polyarticular vs systemic juvenile idiopathic arthritis. *Autoimmunity Reviews*.

[12] Pascual V, Allantaz F, Arce E, Punaro M, Banchereau J (2005). Role of interleukin-1 (IL-1) in the pathogenesis of systemic onset juvenile idiopathic arthritis and clinical response to IL-1 blockade. *Journal of Experimental Medicine*.

[13] Prakken B, Albani S, Martini A (2011). Juvenile idiopathic arthritis. *The Lancet*.

[14] Mellins ED, MacAubas C, Grom AA (2011). Pathogenesis of systemic juvenile idiopathic arthritis: some answers, more questions. *Nature Reviews Rheumatology*.

[15] De Jager W, Hoppenreijs EPAH, Wulffraat NM, Wedderburn LR, Kuis W, Prakken BJ (2007). Blood and synovial fluid cytokine signatures in patients with juvenile idiopathic arthritis: a cross-sectional study. *Annals of the Rheumatic Diseases*.

[16] De Benedetti F, Massa M, Robbioni P, Ravelli A, Burgio GR, Martini A (1991). Correlation of serum interleukin-6 levels with joint involvement and thrombocytosis in systemic juvenile rheumatoid arthritis. *Arthritis & Rheumatism*.

[17] Foell D, Roth J (2004). Proinflammatory S100 proteins in arthritis and autoimmune disease. *Arthritis & Rheumatism*.

[18] Quartier P, Allantaz F, Cimaz R (2011). A multicentre, randomised, double-blind, placebo-controlled trial with the interleukin-1 receptor antagonist anakinra in patients with systemic-onset juvenile idiopathic arthritis (ANAJIS trial). *Annals of the Rheumatic Diseases*.

[19] Lequerré T, Quartier P, Rosellini D (2008). Interleukin-1 receptor antagonist (anakinra) treatment in patients with systemic-onset juvenile idiopathic arthritis or adult onset Still disease: preliminary experience in France. *Annals of the Rheumatic Diseases*.

[20] Gattorno M, Piccini A, Lasigliè D (2008). The pattern of response to anti-interleukin-1 treatment distinguishes two subsets of patients with systemic-onset juvenile idiopathic arthritis. *Arthritis & Rheumatism*.

[21] Nigrovic PA, Mannion M, Prince FHM (2011). Anakinra as first-line disease-modifying therapy in systemic juvenile idiopathic arthritis: report of forty-six patients from an international multicenter series. *Arthritis & Rheumatism*.

[22] Goldbach-Mansky R, Dailey NJ, Canna SW (2006). Neonatal-onset multisystem inflammatory disease responsive to interleukin-1*β* inhibition. *The New England Journal of Medicine*.

[23] Ruperto N, Quartier P, Wulffraat N (2012). A phase II study to evaluate dosing and preliminary safety and efficacy of canakinumab in systemic juvenile idiopathic arthritis with active systemic features. *Arthritis & Rheumatism*.

[24] Lovell DJ, Giannini EH, Kimura Y (2009). Long-term safety and efficacy of rilonacept in patients with systemic juvenile idiopathic arthritis (SJIA). *Arthritis & Rheumatism*.

[25] De Benedetti F, Martini A (1998). Is systemic juvenile rheumatoid arthritis an interleukin 6 mediated disease?. *The Journal of Rheumatology*.

[26] Fonseca JE, Santos MJ, Canhão H, Choy E (2009). Interleukin-6 as a key player in systemic inflammation and joint destruction. *Autoimmunity Reviews*.

[27] Fishman D, Faulds G, Jeffey R (1998). The effect of novel polymorphisms in the interleukin-6 (IL-6) gene on IL-6 transcription and plasma IL-6 levels, and an association with systemic-onset juvenile chronic arthritis. *Journal of Clinical Investigation*.

[28] Ogilvie EM, Fife MS, Thompson SD (2003). The -174G allele of the interleukin-6 gene confers susceptibility to systemic arthritis in children: a multicenter study using simplex and multiplex juvenile idiopathic arthritis families. *Arthritis & Rheumatism*.

[29] Yokota S, Imagawa T, Mori M (2008). Efficacy and safety of tocilizumab in patients with systemic-onset juvenile idiopathic arthritis: a randomised, double-blind, placebo-controlled, withdrawal phase III trial. *The Lancet*.

[30] De Benedetti F, Brunner HI, Ruperto N (2010). Efficacy and safety of tocilizumab in patients with systemic juvenile idiopathic arthritis (sJIA): 12-week data from the phase 3 TENDER trial. *Annals of the Rheumatic Diseases*.

[31] Maeno N, Takei S, Nomura Y (2002). Highly elevated serum levels of interleukin-18 in systemic juvenile idiopathic arthritis but not in other juvenile idiopathic arthritis subtypes or in Kawasaki disease. *Arthritis & Rheumatism*.

[32] Kawashima M, Yamamura M, Taniai M (2001). Levels of interleukin-18 and its binding inhibitors in the blood circulation of patients with adult-onset Still's disease. *Arthritis & Rheumatism*.

[33] De Jager W, Vastert SJ, Beekman JM (2009). Defective phosphorylation of interleukin-18 receptor *β* causes impaired natural killer cell function in systemic-onset juvenile idiopathic arthritis. *Arthritis & Rheumatism*.

